# Assets among low-income families in the Great Recession

**DOI:** 10.1371/journal.pone.0192370

**Published:** 2018-02-05

**Authors:** Valentina Duque, Natasha V. Pilkauskas, Irwin Garfinkel

**Affiliations:** 1 Population Studies Center and Department of Economics, University of Michigan, Ann Arbor, MI, United States of America; 2 Gerald R. Ford School of Public Policy, University of Michigan, Ann Arbor, MI, United States of America; 3 School of Social Work, Columbia University, New York, NY, United States of America; Universidad Veracruzana, MEXICO

## Abstract

This paper examines the association between the Great Recession and real assets among families with young children. Real assets such as homes and cars are key indicators of economic well-being that may be especially valuable to low-income families. Using longitudinal data from the Fragile Families and Child Wellbeing Study (N = 4,898), we investigate the association between the city unemployment rate and home and car ownership and how the relationship varies by family structure (married, cohabiting, and single parents) and by race/ethnicity (White, Black, and Hispanic mothers). Using mother fixed-effects models, we find that a one percentage point increase in the unemployment rate is associated with a -0.5 percentage point decline in the probability of home ownership and a -0.7 percentage point decline in the probability of car ownership. We also find that the recession was associated with lower levels of home ownership for cohabiting families and for Hispanic families, as well as lower car ownership among single mothers and among Black mothers, whereas no change was observed among married families or White households. Considering that homes and cars are the most important assets among middle and low-income households in the U.S., these results suggest that the rise in the unemployment rate during the Great Recession may have increased household asset inequality across family structures and race/ethnicities, limiting economic mobility, and exacerbating the cycle of poverty.

## Introduction

The Great Recession that officially began in December 2007 and ended in June 2009 was the worst recession since the Great Depression [1]. The unemployment rate rose from 5 percent in December 2007 to 10.1 percent in October 2009, and housing prices plummeted by 20% [2, 3]. The simultaneous collapse in the labor, housing, and stock markets, and the slow economic recovery resulted in many families experiencing a dramatic overall decline in net worth [4, 5]. Although a number of studies have documented asset loss in the Great Recession, far less research has examined whether asset ownership changed among lower-income populations or among families with children, those who might be most vulnerable. Using a longitudinal dataset of low-income families with children, we explore whether the Great Recession affected asset ownership and differences by race/ethnicity and family structure.

Our study focuses on lower-income urban families with young children. Although we know that the Great Recession hit more economically vulnerable populations hardest [6], no research has examined how the recession affected asset ownership among lower income families with children. The economic impact and ensuing instability that comes with home or car loss may be particularly pronounced for families with children. Research on the Great Depression showed that children in families that were affected by the recession had poorer outcomes as adults [7, 8]. Additionally, because many low-income families have limited access to formal credit markets and have low levels of savings, they may be more likely to draw upon their assets in poor economic times to help smooth consumption than higher income families who may draw upon other assets (such as stocks or bonds; [9–11]). Thus, these families may be most vulnerable to losing the few assets they have.

This paper examines the link between the unemployment rate and home and car ownership among lower-income families with children. We study these two assets because they represent the two most commonly owned assets for low and middle income families in the U.S. [12, 13] and are closely linked with wellbeing. Prior research documents that many families lost their homes in the Great Recession [14, 15], but no research has examined home ownership among families with children. This is a large oversight as home ownership and housing stability are associated with many positive outcomes for families and children [16].

Little research examines what happened to car ownership in the Great Recession and none has explored the effects on low-income families or those with children. For lower income families, cars represent their largest asset and provide access to employment, services, and other important opportunities [11, 17]. Thus, the loss of a car could represent a huge adverse shock to the economic wellbeing of low-income families. We add to the literature by examining home and car loss separately among lower-income families with children.

We also study differences in the link between unemployment and car and home ownership by key demographic characteristics: family structure (single, cohabiting, married) and race/ethnicity (Black, White, Hispanic). Although we know that racial and ethnic disparities in assets widened considerably in the years between 2007 and 2010 [18, 19], less is known about racial/ethnic differences in car ownership, nor how these changes affected families with children. The literature exploring differences in the effect of the Great Recession on asset holding by family structure is very limited. This is an important oversight as today, more than 40% of children are born to unwed parents, and these families may have been at even greater risk during the Great Recession [20, 21]. Research documents children’s “diverging destinies” during the second demographic transition; children from more highly educated, married families are gaining resources, whereas those from less educated, cohabiting, and single families have fewer [22]. By studying whether there were differences in the effects of the Great Recession on asset ownership by family structure (as well as race/ethnicity), we can better understand if the recession exacerbated or ameliorated these trends in economic wellbeing by family structure.

We use the first five waves of the Fragile Families and Child Wellbeing Study (FFCWS) to study whether the unemployment rate is associated with home and car ownership among families with young children and differences by demographic characteristics. These data are well suited to address many of the shortcomings of existing work. Previous studies have mostly measured the effects of the Great Recession by comparing the mean level of household assets in 2007 with the mean level in 2009 using cross section data [3, 23, 24]. Although informative, these comparisons fail to account for a number of factors associated with a household’s capacity to accumulate assets over time and their propensity to live in an area with high unemployment, which we can address by using longitudinal data with person specific fixed-effects. Second, unlike some prior research, which relies on measures of individual unemployment, we use the city level unemployment rate, which is exogenous to the individual. Third, because the FFCWS oversampled non-marital births, families in the study are relatively economically disadvantaged and there is ample variation by family structure (allowing us to compare “fragile” or unmarried families to married families) and race/ethnicity, two demographic characteristics that are closely linked with economic and asset stratification [25]. Last, our study spans 10 years, including both the Dot Com recession in the early 2000s and the onset of the Great Recession (the latest wave of data collection occurred from 2007 to 2010), providing us with significant variation in the local area unemployment rate over time to help precisely estimate the effect of a change in the unemployment rate on a household’s assets.

We begin by describing the literature on asset accumulation among low-income families and differences between car and home ownership. We then review the theory and literature relating to the unemployment rate and asset ownership. Last, we consider the theoretical reasons to expect differences in the effect of the Great Recession on asset ownership by family structure and race/ethnicity.

## Background

### Assets among low-income households

Asset accumulation among low-income households follows two key patterns: first, low-income households accumulate very little in the way of liquid assets [26, 27] and second, when low-income households are able to accumulate assets, they tend to do so in the form of real assets from which they also derive consumption [28]. In particular, low-income households tend to buy cars and homes [12, 13, 17, 19, 28–33].

Home and car ownership provide families with concrete resources and are linked with other positive economic outcomes [34, 35]. Home ownership, in addition to providing physical shelter, is associated with economic stability, security, safety, and better health and life satisfaction among adults [16, 36] and for children, housing stability is positively linked with their healthy development [37–41]. Car ownership provides individuals with the ability to find and travel to employment and to work more hours [13, 32, 42–44]. For certain economically disadvantaged groups (single-mothers with low levels of education), vehicles frequently represent their largest asset [11] and may be advantageous for safety reasons or getting to work when working non-standard work hours [45].

In addition to having different benefits, cars and homes also function differently as assets. First, vehicles are more liquid than homes, making them a more efficient means of buffering against negative income shocks. However, vehicles depreciate over time, whereas homes may appreciate. Last, because buying a home generally requires a large upfront financial investment and a long-term commitment as compared to cars, even among lower income households those who can afford a home are likely to be better off.

Previous research has shown that key demographic characteristics like age, race/ethnicity, education, income, and marital status predict home and car ownership [46–49]. For low-income families, institutional mechanisms and government policies can create particularly large barriers to ownership (e.g., access to formal credit markets; [10, 11, 26]). This is especially true for minority families, where discrimination in home lending and car financing can reduce asset ownership [50]. Research has also found that low language proficiency and legal status reduces asset ownership [51]. For these reasons, we also know that asset ownership varies by race/ethnicity.

### Unemployment and assets

There are a number of ways in which changes in the local area unemployment rate might affect home and car ownership. Families that live in areas of high unemployment are at a higher risk of job loss, reduced wages, or decreased working hours. This in turn affects the capacity of households to save and accumulate assets, as well as their ability to retain assets they already own. High unemployment may also affect families’ social support networks as networks tend to be homophilous; if one family in a network is hit by unemployment many are likely to be hit [52–54] [55]. Thus, in an economic crisis, a family may not be able to rely on friends or other family members to make a home or car payment, putting them at greater risk of asset loss. Moreover, high local unemployment rates may lead to worse physical and mental health that may affect an individual’s productivity putting them at a higher risk of job loss [56].

There are a number of recent studies exploring the effects of the Great Recession on household economic outcomes. Most of these studies have focused on wealth as opposed to assets [19, 23] and on the heterogeneous effects by race/ethnicity, age, and education [19, 57–61]. The research focusing on asset ownership (especially car ownership) is more limited [15, 19, 62, 63] and less is known about differences across groups, especially family structure. To our knowledge only two studies examined car ownership, which found little change in the total number of vehicle owners between 2005 and 2009, nor changes by race/ethnicity [19, 24]. These studies did not examine changes in car ownership among low-income families, for whom it may be particularly consequential, nor did they examine families with children. Moreover, existing studies exploring changes in home and car ownership have mostly provided comparisons in means before and after the Great Recession. We extend this earlier research by using a more rigorous methodological approach (mother fixed-effects) and an exogenous measure of unemployment in a population that has largely been unstudied: low-income families with children.

### Heterogeneity by family structure

The association between the local unemployment rate and asset ownership may vary by nuclear family structure. First, marital status is highly correlated with economic wellbeing. Married mothers are much better off economically as compared to unmarried mothers. For example, among families with children who were headed by a single mother, 40% were poor, as compared to only 7% of children in married households in 2003 [64]. Because single mothers are much more likely to be low-income, and low-income households were much more likely to experience unemployment during the Great Recession [65], we might anticipate single mothers to also experience the greatest loss in assets, followed by cohabiting mothers and married mothers to experience the smallest losses.

Second, we know from prior literature that married households are more likely to accumulate assets than unmarried households [49, 66–69]. Marriage (and cohabitation to a lesser extent) is a wealth promoting institution (through economies of scale, or improvements in health, dual earners; [70–72]. Single parents, on the other hand, face the dual roles of provider and caregiver, and fewer economies-of-scale, and therefore may have more difficulty accumulating savings and other assets over time [73]. Because married mothers have greater assets than unmarried mothers, this suggests three possible effects of the recession. First, because they have more assets besides their cars and homes, married mothers may smooth consumption in a recession by selling other assets, making them less likely to lose their home or car than unmarried mothers. Yet, second, married mothers are also much more likely to own homes and cars as compared to single or cohabiting mothers. A mother cannot lose an asset she does not own, thus, married mothers may experience more home and car loss than unmarried mothers. Last, single or cohabiting mothers who do own assets, may be quite different from other unmarried mothers, as they have managed to accumulate sufficient wealth to own those assets. Thus, we may not find differences in car and home ownership by family structure. We examine these three hypothetical directions by studying differences by family structure.

Third, we might expect married and cohabiting couples to differ from single mothers as they include two adult household members, and thus, two potential wage earners. In an economic crisis, married and cohabiting households may be able to adjust their labor supply (or labor force participation) to offset job-related problems faced by a partner. Prior research has found that married couples adjust their labor supply when facing an economic or health problem [67, 70, 74–76]. Cohabitation, however, is generally a less stable relationship, where expectations and financial obligations of each partner are more uncertain, and where legal enforcement may be more difficult [77]. This instability in the cohabiting relationship may result in less risk sharing between partners. Even among couples who transition from cohabitation into marriage, cohabitation may alter rates of consumption, saving, and investing, setting cohabiting households on distinct trajectories from married households that influence asset accumulation even after the transition to marriage and potentially affecting responses to economic crises [78].

In sum, although there are some reasons to expect the assets of married families to be most strongly affected in a recession, there are more reasons to anticipate that married households will lose the fewest assets, followed by cohabiting couples, with single parents faring worst.

To date, we are only aware of one study that has investigated how wealth changed in the Great Recession by relationship status. Pfeffer and coauthors (2013)[61] estimated the impact of the Great Recession on wealth by studying changes in household net worth over time (from 2007 to 2011), holding other household demographic and socioeconomic characteristics constant. Their study is of note as they investigated heterogeneity in the changes in wealth by relationship status (married versus single) and by whether a child was present in the household. However, they did not study differences by relationship status among families with children, nor did they study home or car loss directly. Pfeffer and colleagues found that married couples had more wealth both before and after the Great Recession than single men and women but despite lower levels of wealth, single households experienced a larger percent loss in wealth during the recession. They also found that households with children had lower levels of wealth (pre and post-recession), but that the percent loss was greater for households with children. We add to the literature by examining differences by family structure in home and car loss. In doing so, we can investigate whether more economically disadvantaged groups were hit harder in the Great Recession, potentially exacerbating inequality by family structure.

### Heterogeneity by race/ethnicity

There are also a number of reasons to expect differences in the association between the local unemployment rate and car and home ownership by race or ethnicity. First, it is well documented that minority households were hit harder by the recession than White households [19, 79]. Second, race/ethnicity is closely linked with income and with family structure. As noted earlier, lower-income groups experienced more unemployment than any other group in the recession, and low-income families are disproportionately Black and Hispanic. Third, as demonstrated above, we anticipate single mothers to experience the greatest home and car losses, followed by cohabiting families. Family structure and race/ethnicity are also highly correlated; in 2013, 72% of Black children were born to unmarried mothers, 53% of Hispanic children and 29% of White children [21]. Rates of cohabitation are also higher among Hispanic unmarried families than Black families [68]. Last, areas of the U.S. with a higher density of Hispanic families were hit harder by the Great Recession [19]. Thus we expect that Hispanic and Black families will have been more likely to lose their cars and homes in the recession than White families.

In sum, this paper is the first to examine the link between the Great Recession and home and car ownership focusing on low-income families with children. We extend beyond earlier research by using more rigorous methodological approaches and by examining car and home ownership, two key assets for low-income families. We are the first to explore differences in asset ownership changes by race/ethnicity and family structure among low-income families with children. By studying differences by these characteristics, we can better consider whether the Great Recession exacerbated or ameliorated economic inequality by race/ethnicity and family structure and whether the recession contributed to children’s “diverging destinies” [22].

## Method

### Data

The Fragile Families and Child Wellbeing Study (FFCWS) is a birth cohort study of 4,898 children born between 1998 and 2001 in 20 large U.S. cities (populations of 200,000 or more). Mothers and fathers were interviewed shortly after the birth and follow-up data were collected one (1999–2001), three (2001–2003), five (2003–2006), and nine years (2007–2010) after the child’s birth (five waves total). The FFCWS study oversampled births to unwed parents, which constituted about three fourths of the total respondents [80]. Thus the FFCWS provides us with ample data to examine differences in the associations by family structure and changes over time. While there are other nationally representative datasets (such as the Survey of Income and Program Participation), which offer richer measures of assets, they are generally limited in their ability to examine differences by family structure, and especially limited if we are interested in examining families with children.

**Fig 1:**
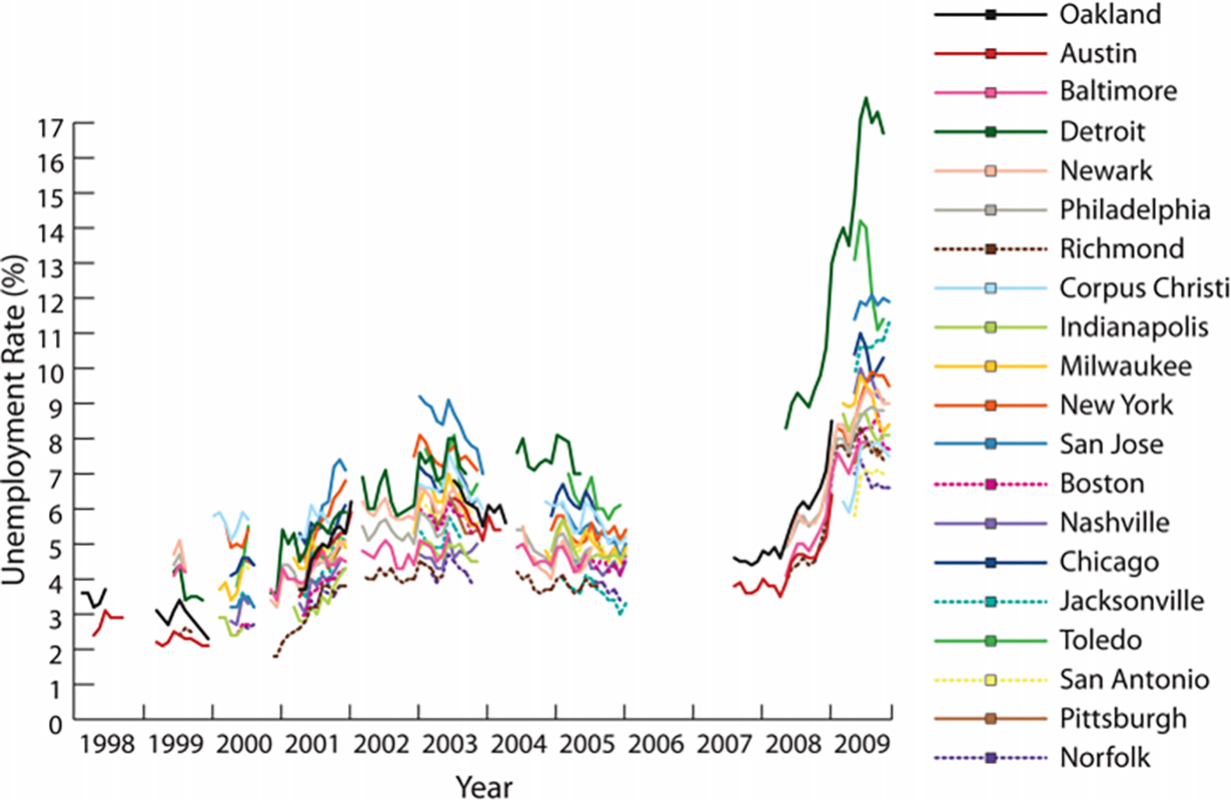
Unemployment rate during interviewing. Source: Pilkauskas, Currie & Garfinkel, (2012)[81].

Fig 1 depicts the considerable variation in the unemployment rate over time and across the twenty metropolitan sample cities in FFCWS. The variation is especially large during the Great Recession years (2007–2009) when the last data collection took place, making the FFCWS dataset particularly suitable for the analysis of the relationship between unemployment and family’s assets. For instance, cities like Detroit or Toledo experienced a rise in the unemployment rate from 7 to 17% and from 6 to 13%, respectively, from 2005 to 2009 while areas such as Richmond or Boston, only experienced 4 and 3 percentage point increases during the same period. We used mother’s reports, as children are more likely to live with their mothers, and because mothers had higher response rates than fathers. Of the almost 5,000 mothers interviewed at baseline, 90% were interviewed at year 1, 88% were re-interviewed at year 3, 87% at year 5, and 76% at year 9.

We pooled the data across survey waves (years 1 to 9) to study the association between the unemployment rate and assets. Of the 4,898 mothers (19,592 mother-year observations) included in FFCWS, 4,767 mothers (19,074 mother-year observations) completed at least one follow up survey after baseline and had complete information on all covariates (we had very few cases of missing information on covariates, approximately 2%). Of these 4,767 mothers we lost 834 cases (3,856 mother-year cases) as they did not provide complete information on home and/or car ownership, so the final sample was reduced to 3,933 mothers (15,732 person-years).

Comparing mothers who attrited (3,856 mother-year cases) with mothers who did not attrite (15,732 mother-year cases), we found that mothers who attrited were more economically disadvantaged. These mothers were less educated, more likely to be cohabiting or single, a minority, an immigrant, or poor at the baseline interview, compared to mothers who remained in the sample, although these differences were small.

### Measures

#### Home and car ownership

Home ownership was defined as a dummy variable that took the value of one when a mother reported owning a home, or if her coresident partner (married or cohabiting) owned a home and zero otherwise (we only study homes in which mothers are living as we do not have information on other potential homes they may own). There are a small number of cases (less than 0.5 percent of the sample) where she lives in a home owned by another person (not her partner), in those cases we did not include her as a homeowner. Car ownership was coded as one when a mother (or partner) reported owning a car and zero otherwise. Our study examines changes in asset ownership, rather than loss per se, as we cannot distinguish a loss (say foreclosure) from the sale of an asset.

#### Unemployment rate

Using data from the Bureau of Labor Statistics’ Local Area Unemployment Statistics (LAUS), we appended a measure of the city unemployment rate to the FFCWS data based the mother’s sample city using her Core Based Statistical Area (CBSA, similar to a Metropolitan Statistical Area) and the date of her interview (at each survey wave). We used the city in which she was originally sampled (rather than her current city of residence) to control for the possibility of endogenous migration in response to changes in unemployment rates. But, because a proportion of mothers migrated from their sample city (about 20%), we also analyzed how the average unemployment rate over the last year in the current city of residence was associated with changes in household assets. These results are discussed in the extensions section.

One may also be concerned that the unemployment rate is not exogenous to the mother if her own unemployment is included in the unemployment rate. However, it is important to note that N’s for any individual city range from 100–350 per city per survey wave and each city in our sample has a population of 200,000 or greater (many are much larger, like New York or Philadelphia), thus we do not believe this is a threat to the exogeneity of the unemployment rate.

#### Family structure

Family structure was defined as being married, cohabiting, or single at the birth of the child. We studied relationship status at the birth (as opposed to later relationship statuses) for two reasons. First, patterns of asset accumulation are likely to vary greatly by relationship status at child’s birth [22]. Second, since family structure can be affected by economic fluctuations, by holding constant the relationship status at birth we reduced the problem of endogenous controls. In the extensions section, we investigate the robustness of our results when we define family structure as time varying.

#### Race/ethnicity

Race/ethnicity was defined as non-Hispanic White, non-Hispanic Black, Hispanic, or other race/ethnicity. Because the other race/ethnicity group is relatively small, our analyses by subgroup are limited to White, Black, and Hispanic families.

#### Control variables

We included as control variables a number of basic socioeconomic and demographic characteristics of the mother that were measured at the baseline survey that research finds are associated with asset accumulation [48]. These variables included: a measure of mother’s age at the birth of the child (coded as less than <20, 20–24, 25–29, 30–34, 35+), a set of dummy variables for education (less than high school, high school, some college, and college or more) and immigrant status (foreign born). We also controlled for the income-to-needs ratio (using the official U.S. poverty thresholds, adjusted by family size and year) to create dummy variables indicating less than 100% of poverty, 100–199% of poverty, 200–399% of poverty, and 400%+ of poverty. Lastly, we included a control for whether the focal child (the child who was born when mothers were sampled for the study) is the first child in a family.

### Analytic strategy

To estimate whether the unemployment rate was related to car or home ownership, we used two empirical models, one that pools data from years 1 through 9 (survey waves t = 1–4) with covariates from the baseline survey (t = 0) and controls for city and wave of interview fixed-effects, and a second one that accounts for time-invariant mother fixed-effects (following a number of studies that use the FFCWS to examine the effects of the Great Recession on various outcomes [56, 82]. For ease of interpretation, we employed linear probability models (as the coefficient can be directly interpreted as a percent change), but using other functional forms such as logit models provided substantively similar estimates [83].

Eq 1 describes the pooled model without mother fixed-effects:

$$Y_{i,c,t}=\beta_{0}+\beta_{1}{UR}_{i,c,t}+\beta_{2}X_{i,t0}+\alpha_{c}+\alpha_{t}+\varepsilon_{i,c,t}$$ (1)

where Y_i,c,t_ denotes the i’th respondent’s home or car ownership measured in baseline city c at time (wave) t, UR is the average unemployment rate over the last year at the baseline city, X is a matrix of mother i characteristics measured at baseline (t = 0). The terms α_c_ and α_t_ are vectors of dummies for baseline city and survey wave, respectively. The city fixed-effects control for time-invariant factors at the city level such as specific characteristics of the local housing market and the wave fixed-effects absorb factors/shocks that occurred at a given time that affected both the labor market conditions and a family’s assets in that particular wave. The term ε is the disturbance. All models were two-way clustered at the baseline city and at the mother level to account for the within-city and within-mother correlation in the observations [84]. The main parameter of interest is β_1_.

The second model controls for mother-specific fixed-effects and is estimated using Eq 2. The term μ_i_ represents a mother’s time-invariant specific effect and the only covariate included in this specification is αt, the survey wave dummy.

$$Y_{i,c,t}=\beta_{1}{UR}_{i,c,t}+\alpha_{t}+\mu_{i}+\varepsilon_{i,c,t}$$ (2)

The mother (or individual) fixed-effects model exploits the longitudinal nature of FFCWS to control for both observed and unobserved time- invariant characteristics of the mother, which may be correlated with both the probability of residing in a city with high unemployment rate and owning few assets. For instance, if a mother belongs to a demographic group that is likely to be particularly affected by unemployment, she may also be less likely to own a home or a vehicle.

To study differences in the association between the unemployment rate and real assets by family structure and race/ethnicity, we performed separate analyses by stratifying the sample into married, cohabiting, and single mothers, and by non-Hispanic Whites, non-Hispanic Blacks, and Hispanics.

## Results

### Descriptive characteristics

Table 1 provides descriptive information for the full sample of families and by family structure and race/ethnicity at child’s birth (t = 0 of the survey). In terms of home and car ownership, which is averaged over time in the pooled data (t = 1, 2, 3, and 4 of the survey), we find that less than one-quarter of the sample owned a home and 66% owned a car. Figures from the Census Bureau in 2014 show that 66% of Americans are homeowners and 95% own a vehicle, which suggests that the FFCWS sample is a particularly economically vulnerable population[85].

**Table 1:** Sample descriptive statistics by relationship status.

	Full	Relationship Status			Race/Ethnicity		
	Sample	Married	Cohabiting	Single	White	Black	Hispanic
*Outcomes*							
Home owner ^a^^,^^b^^,^^c^ ^j^^,^^l^^,^^m^	0.24	0.55	0.17	0.10	0.50	0.18	0.22
Car owner ^a^^,^^b^^,^^c^ ^j^^,^^l^^,^^m^	0.66	0.89	0.66	0.50	0.88	0.55	0.66
*Mother characteristics*							
Relationship Status:							
Married ^j^^,^^l^^,^^m^	0.25	1.00	-	-	0.48	0.13	0.23
Cohabitating ^j^^,^^l^^,^^m^	0.36	-	1.00	-	0.31	0.34	0.46
Single ^j^^,^^l^^,^^m^	0.39	-	-	1.00	0.20	0.53	0.32
Race/Ethnicity:							
White ^a^^,^^b^	0.23	0.46	0.19	0.11	1.00	-	-
Black ^a^^,^^b^^,^^c^	0.48	0.24	0.45	0.66	-	1.00	-
Hispanic ^a^^,^^b^^,^^c^	0.26	0.24	0.33	0.21	-	-	1.00
Other ^a^^,^^b^	0.03	0.07	0.03	0.02	-	-	-
Immigrant ^a^^,^^b^^,^^c^ ^l^^,^^m^	0.15	0.23	0.16	0.08	0.05	0.05	0.41
Age:							
<20 ^a^^,^^b^^,^^c^ ^j^^,^^l^^,^^m^	0.17	0.03	0.18	0.25	0.13	0.20	0.17
20–24 ^a^^,^^b^^,^^c^ ^j^^,^^l^	0.36	0.18	0.42	0.40	0.28	0.38	0.40
25–29 ^a^^,^^b^ ^j^^,^^l^^,^^m^	0.23	0.31	0.22	0.20	0.24	0.23	0.22
30–34 ^a^^,^^b^ ^j^^,^^l^	0.14	0.29	0.10	0.09	0.16	0.10	0.11
> = 35 ^a^^,^^b^ ^j^^,^^l^	0.09	0.19	0.07	0.06	0.16	0.07	0.07
Education:							
Less than HS ^a^^,^^b^^,^^c^ ^j^^,^^l^^,^^m^	0.33	0.14	0.38	0.41	0.19	0.34	0.51
HS ^a^^,^^b^ ^j^^,^^l^^,^^m^	0.30	0.19	0.34	0.34	0.26	0.36	0.25
Some college ^a^^,^^b^ ^j^^,^^l^^,^^m^	0.25	0.29	0.24	0.22	0.28	0.25	0.20
College or more ^a^^,^^b^^,^^c^ ^j^^,^^l^^,^^m^	0.12	0.37	0.03	0.03	0.28	0.05	0.04
Income-to-needs ratio:							
0–100 ^a^^,^^b^^,^^c^ ^j^^,^^l^^,^^m^	0.40	0.13	0.42	0.55	0.15	0.41	0.36
100–199 ^a^^,^^c^ ^j^^,^^l^	0.25	0.18	0.30	0.24	0.19	0.24	0.26
200–399 ^a^^,^^b^^,^^c^ ^j^^,^^l^	0.14	0.22	0.14	0.09	0.17	0.13	0.11
400+ ^a^^,^^b^^,^^c^ ^j^^,^^l^^,^^m^	0.18	0.44	0.10	0.07	0.38	0.10	0.10
First birth ^a^^,^^c^ ^j^^,^^l^	0.38	0.35	0.36	0.43	0.46	0.33	0.39
N–mother-years	15,732	3,905	5,634	6,138	3,438	7,637	4,078
N–mothers	4,898	1,187	1,782	1,929	1,029	2,336	1,335

Note: HS = High school. Statistically significant differences from t-tests (*p*<0.05) are noted as follows:

^a^ married versus cohabiting mothers

^b^ married versus single mothers

^c^ cohabiting versus single mothers.

^j^ White versus Black mothers

^l^ Black versus Hispanic mothers

^m^ White versus Hispanic mothers.

In terms of other demographic characteristics, approximately 25% of the sample was married, 36% cohabiting, and 39% single at the birth of the child, providing ample sample to study differences by family structure. Similarly, the sample is very racially diverse; 22% of mothers were White, half were Black, and one quarter were Hispanic. Mothers were also relatively economically disadvantaged; 33% had less than a high school degree, whereas only 12% had completed college or more education and 40% of the mothers were poor (income-to-needs ratio below 100) with another quarter being near poor (income-to-needs ratio between 100 and 199).

Differences by family structure reveal that home and car ownership varied enormously by relationship status and race/ethnicity. More than 50% of married households owned a home while 17% and 10% of cohabiting and single parent families did likewise. In terms of car ownership, married mothers were also more likely to own a car (89%) than unmarried households (66% among cohabiting couples and 50% among single parent households). Differences by race/ethnicity show that 50% of White mothers owned a home and 88% owned a car whereas these numbers were significantly lower for Hispanic families (22% owned a home and 66% owned a vehicle) and even lower for Black mothers (18% owned a home and 55% owned a vehicle).

Maternal demographic characteristics also varied by family structure and race/ethnicity. Married mothers were significantly older, more likely to be White, to be an immigrant, were more educated, and had higher income-to-needs ratios than cohabiting or single mothers. Differences by race/ethnicity also reveal that White mothers were significantly wealthier and more educated than non-White mothers.

### Is the unemployment rate linked with home and car ownership?

Table 2 presents the results of the multivariate regression analyses studying the association between the city unemployment rate and car/home ownership for the full sample using a linear regression model without (described in Eq 1) and with individual fixed-effects (described in Eq 2). Results from the specification without individual fixed-effects (Eq 1) are shown in Columns 1 and 3 and analyses with individual fixed-effects (Eq 2) are in Columns 2 and 4.

**Table 2:** The association between the unemployment rate and home and car ownership.

	Home Ownership		Car Ownership	
	OLS	FE	OLS	FE
	(1)	(2)	(3)	(4)
Unemployment rate	-0.006**	-0.005**	-0.005	-0.007***
	[0.003]	[0.002]	[0.004]	[0.003]
Relationship Status				
Cohabiting	-0.140***		-0.070***	
	[0.013]		[0.016]	
Single	-0.175***		-0.173***	
	[0.014]		[0.018]	
Age				
<20	-0.117***		-0.021	
	[0.026]		[0.030]	
20–24	-0.154***		0.027**	
	[0.017]		[0.011]	
25–29	-0.117***		0.071***	
	[0.020]		[0.023]	
30–34	-0.078***		0.013	
	[0.016]		[0.014]	
Race/Ethnicity				
Black	-0.090***		-0.108***	
	[0.011]		[0.012]	
Hispanic	-0.026		-0.007	
	[0.015]		[0.017]	
Other	-0.097***		-0.055**	
	[0.027]		[0.024]	
Immigrant	0.024		0.069***	
	[0.022]		[0.009]	
Education				
Less than HS	-0.194***		-0.141***	
	[0.025]		[0.026]	
HS	-0.167***		-0.048**	
	[0.021]		[0.018]	
Some college	-0.130***		0.007	
	[0.024]		[0.020]	
Income to needs ratio				
0–100	-0.306***		-0.342***	
	[0.014]		[0.020]	
100–199	-0.242***		-0.119***	
	[0.015]		[0.019]	
200–399	-0.133***		-0.026*	
	[0.019]		[0.014]	
First birth	-0.030***		-0.018**	
	[0.010]		[0.009]	
N—mother-years	15,677	15,677	15,716	15,716
N—changers		946		1,677
% change in outcome due to a 5pp increase in UR	-12.5%	-10.4%	-3.8%	-5.3%

Note: OLS = ordinary least squares. FE = individual fixed-effects. i) The sample is pooled and includes all mothers in waves 2–5 who report information on assets; ii) Models in columns 1 and 3 control for mother individual characteristics, baseline city and wave fixed-effects, and errors are clustered at the baseline city and mother levels (see Eq 1); iii) Models in columns 2 and 4 control for wave fixed-effects and mother specific fixed-effects (see Eq 2); iv) Standard errors are shown in brackets; v) The predicted percent change in home and car ownership is calculated based on a 5 percentage point increase in the unemployment rate (UR) in year 9 (wave 5).

*** p<0.01

** p<0.05

* p<0.1

We found that the unemployment rate was associated with a decrease in home ownership in the analyses without individual fixed-effects: a one percentage point increase in the unemployment rate was associated with a 0.6 percentage point lower home ownership (or a 2.5% decline with respect to the mean). Controlling for individual fixed-effects (Eq 2) provided estimates that were remarkably similar in size to those obtained from the linear models (0.5 percentage points lower home ownership or 2.1% decline with respect to the mean), but were more precisely estimated. The individual fixed-effects estimates confirmed that as the economy worsened, home ownership declined. For car ownership we found similar results to those for home ownership, although the analyses without individual fixed-effects were not statistically significant. In comparison, the regressions with individual fixed-effects showed that a one-percentage point increase in the unemployment rate was associated with significantly lower levels of car ownership (-0.7 percentage points or -1.1% with respect to the mean).

To investigate how the Great Recession affected home and car ownership we conducted a simulation in which we estimated the change in car ownership when the unemployment rate was 5% as compared to when it was 10%, akin to the change that occurred during the Great Recession (between 2007 and 2010). Because our modeling approach examined how a one-percentage point change in the unemployment rate was associated with a change in home ownership or car ownership, we examined a 5-percentage point change as a way to model what happened in the Great Recession. The results of the simulation are shown in the last line of Table 2. The findings indicate that when the unemployment rate increased by 5 percentage points, families were 10.4% less likely to own their home and 5.3% less likely to own a car.

In terms of the covariates in the full regression models, we found that cohabitation was associated with a 14 percentage points (pp) lower probability of home ownership relative to being married, while single parent households were 18pp less likely to own a home relative to married families. Net of other factors, Black mothers were 9pp less likely to own their home compared to White mothers, whereas Hispanic mothers were 2.6pp less likely (this difference was not statistically significant). Moreover, we see that the covariates suggest highly non-linear effects of income on home and car ownership, which is well documented in previous research [67, 86]. For example, we found that having an income-to-needs ratio of 0–100 was associated with 31pp lower home ownership compared to the wealthiest group, whereas having an income-to needs-ratio between 100 and 199 or between 200–399 was associated with 24pp and 13pp lower probability of home ownership, respectively.

### Do the associations vary by family structure?

Given the heterogeneous distribution in asset holdings across family structure, we also explored whether the association between the unemployment rate and home and car ownership differed by family structure. In Table 3 we ran the same analyses on home and car ownership stratifying the sample by mother’s relationship status at the child’s birth. We found that an increase in the unemployment rate significantly reduced home and car ownership among unmarried households, but was not associated with changes in asset ownership among married households (the coefficients for married couples are both small and not significant). Results are mostly consistent across specifications (Eqs 1 and 2, with and without fixed-effects), although they tend to be more precisely estimated when we employ the mother fixed-effects model. We found that an increase in the unemployment rate significantly decreased the likelihood of owning a home among cohabiting couples (by 0.7pp using Eq 2), but there was no association for car ownership. In comparison, we found that the unemployment rate was associated with a small decline in single mothers’ home ownership (although it was not statistically significant in the fixed-effects specification, Eq 2), and the unemployment rate was significantly associated with a 1.5pp decline (or a 3% fall with respect to the mean when including fixed-effects) in the probability of car ownership among single mothers (both models provided similar estimates).

**Table 3:** The association between the unemployment rate and home and car ownership by relationship status and race/ethnicity.

	Home Ownership		Car Ownership	
	OLS	FE	OLS	FE
	(1)	(2)	(3)	(4)
Married				
Unemployment rate	-0.002	-0.003	-0.002	-0.004
	[0.006]	[0.005]	[0.004]	[0.004]
N—mother-years	3,905	3,905	3,942	3,942
N—changers		326		195
% change^1^	-1.8%	-2.7%	-1.1%	-2.2%
Cohabiting				
Unemployment rate	-0.006	-0.007**	0	0
	[0.004]	[0.004]	[0.005]	[0.004]
N—mother-years	5,634	5,655	5,727	5,748
N—changers		349		686
% change	-17.6%	-20.6%	0.0%	-0.0%
Single				
Unemployment rate	-0.009*	-0.004	-0.011*	-0.015***
	[0.004]	[0.003]	[0.006]	[0.005]
N—mother-years	6,138	6,166	6,047	6,075
N—changers		271		796
% change	-34.5%	-20.0%	-11.0%	-15.0%
White				
Unemployment rate	-0.004	-0.008	0.003	0
	[0.006]	[0.005]	[0.003]	[0.004]
N—mother-years	3,437	3,438	3,434	3,435
N—changers		326		195
% change	-3.6%	-7.3%	1.7%	-0.0%
Black				
Unemployment rate	-0.004	-0.001	-0.010*	-0.012***
	[0.004]	[0.003]	[0.005]	[0.004]
N—mother-years	7,599	7,637	7,552	7,590
N—changers		349		686
% change	-3.6%	-3.50%	-11.1%	-33.3%
Hispanic				
Unemployment rate	-0.014**	-0.009**	0	0.002
	[0.005]	[0.004]	[0.004]	[0.005]
N—mother-year	4,068	4,078	4,155	4,165
N—changers		271		796
% change	-31.8%	-20.5%	0.0%	1.5%

Note: OLS = ordinary least squares. FE = individual fixed-effects. i) The sample is pooled and includes all mothers in waves 2–5 who report information on assets; ii) Models in columns 1 and 3 control for mother individual characteristics, baseline city and wave fixed-effects, and errors are clustered at the baseline city and mother levels (see Eq 1); iii) Models in columns 2 and 4 control for wave fixed-effects and mother specific fixed-effects (see Eq 2); iv) Standard errors are shown in brackets.

^1^ The predicted percent change in home and car ownership is calculated based on a 5 percentage point increase in the unemployment rate (UR) in year 9 (wave 5).

*** p<0.01

** p<0.05

* p<0.1

### Do the associations vary by race/ethnicity?

In Table 3, we also investigate whether the association between the unemployment rate and owning a car and/or home varies by race/ethnicity. Two main patterns emerged from these analyses: i) an increase in the unemployment rate was associated with a lower likelihood of owning assets (cars or homes) for Black and Hispanic families and ii), an increase in the unemployment rate did not significantly change asset ownership for White families. A one percentage point increase in unemployment was associated with a -1.2pp using Eq 2/fixed-effects (or a 2.2% with respect to the mean) decrease in car ownership for Black families and a -0.9pp also using Eq 2/fixed-effects (or a 4.1%) decline in home ownership for Hispanics.

### Extensions

#### Migration

Since a number of mothers moved from their original/baseline cities to other areas (19% of mothers did so since the baseline interview), we examined whether economic conditions in the current place of residence, rather than in the sample city, were also associated with home and car ownership. So for example, if a mother was first interviewed in New York, NY, but she later moved to Corpus Christi, TX, in the first unemployment measure (sample city) we appended the unemployment rate for New York for all survey waves. In the second unemployment measure (current city) we appended the New York unemployment rate to the survey waves for when she lived in New York, and then appended the unemployment rate for Corpus Christi for the survey waves in which she resided in Corpus Christi. Although this approach allowed us to study the unemployment rate currently faced by mothers, using the current city of residence unemployment rate is endogenous, as families may self-select to move to cities with better economic conditions. For this reason, the sample city unemployment rate is our preferred model (and used in all of the prior our analyses). Results presented in S1 Table show substantively similar estimates of the unemployment rate in the current city of residence to those obtained using the baseline/original city and similar findings for differences by subgroup, suggesting little selective migration.

#### Testing whether the associations were different during the Great Recession years

Because our model examined the association between the unemployment rate over a 10-year period and asset ownership, we also explored whether the associations changed if we focused on the years of the Great Recession. To do this, we included an interaction between the unemployment rate and a dummy for the Great Recession years. We found no evidence to suggest that the effect of unemployment during the Great Recession was different from other years except for its high unemployment rate. This result is consistent with previous research studying the link between the Great Recession and other outcomes [87].

#### Other measures of labor market conditions

Our main focus in this paper was on the unemployment rate. However, since unemployed workers who grow discouraged in their job search and do not actively participate in the labor market are not officially counted as unemployed, reductions in the unemployment rate may sometimes overstate improvements in the labor market. Alternatively, the unemployment rate may remain high even as employment is rising if discouraged workers come back to the labor market. Thus, in order to more adequately capture fluctuations in the labor market, we also investigated how the city employment-to-population ratio (ER) has affected household’s assets. The ER is defined as the number of employed workers as a proportion of the total population aged 18–64 in a given city. We constructed an annual average ER measure using employment data from the LAUS that come from the Current Population Survey[88], and we appended it to FFCWS data based on a mother’s baseline city of residence and her date of interview.

When we use the ER we get qualitatively similar estimates to those obtained from the unemployment rate, although they did not reach statistical significance. Overall we found that a one percentage point increase in the employment rate was associated with a 1.0pp increase in the probability of home ownership (compared with a -0.5pp decline when we used the unemployment rate) and a 2pp increase in the rise in car ownership (versus a -0.7pp).

#### Other proxies of the Great Recession

The Great Recession was in part prompted by a collapse in the housing market. To test whether our findings held when we examined alternative proxies of the Great Recession, we included a measure of the foreclosure rate. Using data from the Mortgage Bankers Association National Delinquency Survey from 1999 to 2010, which we appended to the FFCWS data at the state-year-month levels, we constructed state-level measures of the percent of all mortgage payment loans that were in foreclosure over the last 12 months. We found that the foreclosure rate was significantly associated with asset ownership and the findings were very similar to those of the unemployment rate. However, when we included both the unemployment and the foreclosure rate in the mother fixed-effects models, we found that although the coefficients remained almost identical, the level of significance declined and in some cases became statistically insignificant, which may be due to insufficient power, or high levels of correlation between the two proxies.

Differences by group showed that the foreclosure rate rather than the unemployment rate was more strongly associated with assets among White households than among minority households, which may be because White families are more likely to own a home and were less likely to experience job loss during the recession as compared to other racial/ethnic groups.

#### Including time-city interactions

The odds of owning a car or a home may vary by geographical location (e.g., public transportation systems varies across cities). Although our OLS models controlled for city fixed-effects, this does not account for within city changes over time. To explore whether changes over time at the local level effected the probability of owing an asset we included city-year interactions in our models, and found that doing so made the relationship between the unemployment rate and home ownership even stronger from 0.6pp to 1pp in the OLS model but for car ownership, the association became smaller, from 0.7pp (baseline model) to 0.3pp, suggesting that changes within cities over time influence home and car ownership in different ways.

#### Interactions between relationship status and race/ethnicity

Family structure and race/ethnicity are highly correlated. Although our main model specifications examined these demographic characteristics separately, we also tested the joint interaction between these two characteristics by conducting mother fixed-effects models stratifying the sample by relationship status and by race/ethnicity (i.e., married and Black, married and White, married and Hispanic). Results from these analyses are shown in S2 Table and were consistent with the main analysis. When the unemployment rate increased, both single mothers and Black mothers were less likely to own a car; hence, as might be expected, the interaction found the strongest association was for Black, single mothers. Similarly, an increase in the unemployment rate was most strongly associated with a decline in home ownership for Hispanic and cohabiting mothers and the interactive model found an even stronger association for Hispanic cohabiting mothers.

#### Heterogeneous effects by pre-recession mortgage debt to household income ratio

One central driver of the Great Recession was the unprecedented credit bubble where many individuals with low creditworthiness received mortgages. Although we cannot directly evaluate mothers’ creditworthiness, in an extension, we examined whether a household’s mortgage payment exceeded or was equal to 30% of annual household income (also known as cost burden in the housing literature or the household leverage ratio). We found that 6.3% of households in our sample were cost burdened and that the effects of the Great Recession on the probability of home ownership for these families was more than three times the average effect of the unemployment rate on the full sample. In comparison, those with lower cost burdens (arguably higher credit worthiness) were half as likely to no longer own their home when the unemployment rate increased as compared to the full sample. These findings contribute to the growing discussion of policies aimed at addressing excessive borrowing [89] and suggest that highly leveraged households are particularly vulnerable in financial crises.

#### Time varying covariates

Our analyses focused on relationship status at the birth of the child as research has found that relationship status at the birth is predictive of disparities in outcomes over time [22] and because the Great Recession could not have affected relationship status at the birth. Yet, if a significant number of families had a relationship transition, we may be misclassifying our groups thereby introducing significant measurement error. Although we argue that baseline relationship is more exogenous than current relationship status, we also investigated whether the results were robust to using a household’s current relationship status.

Results were qualitatively similar to those obtained in the baseline relationship status models when we used time varying measures of relationship status; however, the coefficients were generally more negative, which could reflect the potential endogeneity between the unemployment rate and family arrangements. For example, we found that a 1 percentage point increase in the unemployment rate was associated with a -1.7pp in the probability of car ownership for single mothers, compared to -0.7pp change when we used baseline relationship status. For cohabiting and married mothers we did not find an association between the unemployment rate and home or car ownership when we controlled for time varying relationship status.

Other potential time varying covariates that may influence family assets are mother’s education [90] and income [91]. Although both of these measures are endogenous, including these time varying mother characteristics did not change our substantive findings.

#### Individual labor market outcomes

The preceding discussion pertains to the relationship between the economic conditions at the local level and household’s assets, an approach that assumes that fluctuations in economic conditions in a city are distributed equally across individuals living in a city. In this section we explore whether families who were more directly exposed to the crisis (i.e., were unemployed), experienced the largest declines in assets. This result provides some evidence on potential mechanisms through which home and car ownership declines; however, it is also problematic as individual unemployment is endogenous to asset accumulation and because estimates can suffer from selection bias as people who choose to leave the labor market may be different in unobserved ways to those who stay employed.

Focusing on mother’s labor market participation and using mother fixed-effects, we found that mothers who were personally unemployed experienced a 0.7pp decline in home ownership (although this result was not statistically significant), and a decline in car ownership by 7.4pp. Results by family structure suggest that married mothers who became unemployed did not experience a change in home ownership; however, they were 3.9pp less likely to own a car. Cohabiting and single mothers, as well as White, Black, and Hispanic women were less likely to own a car (by 5 to 8pp) if they became unemployed. We also tested models that accounted for a mother’s individual unemployment as an additional control in our main regression model. We found little change in the city unemployment rate coefficient when controlling for individual unemployment, which provides some evidence that the effect of the Great Recession on assets is not exclusively operating through a mother’s labor force participation.

#### Other functional forms

Since we do not know the exact functional form of how a change in the unemployment rate is related to asset ownership, we explored: i) the presence of non-linearities in the unemployment rate and ii) whether a lagged realization of the unemployment rate changed the association with family’s assets. We found little evidence of non-linearities or lags.

## Discussion

This paper adds to earlier literature examining asset ownership in the Great Recession by studying a particularly vulnerable group, families with young children, exploring car and home ownership separately, assets most likely to be owned by lower income families, and using a more rigorous methodological approach (mother fixed-effects) than prior research, to eliminate any time invariant differences between mothers that might bias our estimates. Overall we found that the Great Recession significantly decreased home and car ownership; a 5 percentage point increase in the unemployment rate was associated with a 10.4% decline (with respect to mean) in home ownership and a 5.3% decline (with respect to mean) in car ownership and our findings were robust to a large set of extensions.

We hypothesized that certain demographic groups, namely minority mothers and unmarried mothers, were likely to be the most vulnerable in the Great Recession, and our results were consistent with those hypotheses. We found that cohabiting and Hispanic mothers were more likely to experience a decline in the probability of home ownership as a result of the Great Recession; whereas Black and single mothers were more likely to no longer own a car. In contrast, we found that married households and White women did not experience a change in these assets during the Great Recession. Thus, our findings suggest, that the Great Recession likely exacerbated economic differences between these groups, rather than closing the gaps.

It is difficult to compare our findings on car ownership or our findings by family structure to prior work as very little research has explored these two questions. However, our results for home ownership by race/ethnicity are consistent with prior research, which found that Hispanic families were particularly likely to experience home loss in the Great Recession [92] as many Hispanic families lived in areas that were strongly affected by the housing real estate collapse [19].

This study has some limitations. Although housing and cars are the most commonly held assets for low-income families, other assets and debts may be more sensitive to changes in the unemployment rate than homes and cars, for instance, savings. This is particularly true for the more economically advantaged families on our study (married families and White families), as we may not be able to observe their asset losses, if say they sell of other liquid assets to avoid home loss. We also cannot say with certainty whether the changes we observed in home and car ownership are losses (e.g., foreclosure) or sales. We might also underestimate the relationship with car ownership as we cannot examine if a family downsizes the number of vehicles they own. Our findings were robust to controlling for within city time trends, but there may be unaccounted factors within cities that change over time and that are associated with both the labor market conditions and asset ownership (such as access to public transportation which might influence mother’s preferences for cars). Last, our sample has limited generalizability, but the oversample of nonmarital births allowed us to distinguish the associations with the local unemployment rate by relationship status.

Despite some limitations, this paper highlights two important policy concerns. First, to the extent that limited asset holdings reduces the potential for a family to achieve social and economic independence, the effects of the unemployment rate accentuated the gap in economic well-being between married and unmarried families, and between White families and non-White families. Second, given the well-documented relationship that exists between households’ assets and child development, the fact that so many families experienced losses could suggest important long-term effects on young children whose parents faced economic distress. In many ways, our findings suggest that the Great Recession exacerbated differences for children who were born to single and cohabiting parents as compared to married parents–perhaps worsening their “diverging destinies”[22]. Future research that examines the Great Recession should take into account differences by family structure, and additionally consider how the Great Recession may have affected children’s mobility and future inequality.

## Supporting information

S1 Table The association between the unemployment rate in a mother’s city of residence and home and car ownership. (DOCX)

S2 Table The association between the unemployment rate and home and car ownership by relationship status and race/ethnicity using mother fixed-effects. (DOCX)
