# Supplementary material for: Assets among low-income families in the Great Recession
Source: PLoS One. 2018 Feb 5;13(2):e0192370. doi: 10.1371/journal.pone.0192370 (PMC5798834; doi:10.1371/journal.pone.0192370)
Supplement: S1 Table — (DOCX) [file pone.0192370.s001.docx]

**S1 Table. The Association between the Unemployment rate in a Mother’s City of Residence and Home and Car Ownership**

|  | Home Ownership | | Car Ownership | |
| --- | --- | --- | --- | --- |
|  | OLS | FE | OLS | FE |
|  | (1) | (2) | (3) | (4) |
| Current unemployment rate | -0.003 | -0.002 | -0.002 | -0.005** |
|  | [0.003] | [0.002] | [0.003] | [0.002] |
| Baseline Unemployment rate | -0.006** | -0.005** | -0.005 | -0.007*** |
|  | [0.003] | [0.002] | [0.004] | [0.003] |
| N - mother-year | 15,677 | 15,726 | 15,716 | 15,765 |

Note: OLS = ordinary least squares. FE = individual fixed-effects. i) The sample is pooled and includes all mothers in waves 2-5 who report information on assets; ii) Models in columns 1 and 3 control for mother individual characteristics, baseline city and wave fixed-effects, and errors are clustered at the baseline city and mother levels (see equation 1); iii) Models in columns 2 and 4 control for wave fixed-effects and mother specific fixed-effects (see equation 2); iv) Standard errors are shown in brackets.

*** p<0.01, ** p<0.05, * p<0.1.
