# Supplementary material for: Assets among low-income families in the Great Recession
Source: PLoS One. 2018 Feb 5;13(2):e0192370. doi: 10.1371/journal.pone.0192370 (PMC5798834; doi:10.1371/journal.pone.0192370)
Supplement: S2 Table — (DOCX) [file pone.0192370.s002.docx]

**S2 Table. The Association between the Unemployment Rate and Home and Car Ownership by Relationship Status and Race/Ethnicity Using Mother fixed-effects.**

|  | Home Ownership | Car Ownership |
| --- | --- | --- |
| Married -White | -0.007 | -0.004 |
|  | [0.007] | [0.004] |
| N - mother-year | 1,720 | 1,724 |
| N - changers | 484 | 483 |
| Married - Black | 0.006 | -0.000 |
|  | [0.011] | [0.010] |
| N - mother-year | 969 | 976 |
| N - changers | 286 | 285 |
| Married - Hispanic | -0.001 | -0.000 |
|  | [0.010] | [0.009] |
| N - mother-year | 940 | 962 |
| N - changers | 291 | 291 |
| Cohabiting - White | -0.013 | 0.004 |
|  | [0.010] | [0.009] |
| N - mother-year | 1,050 | 1,056 |
| N - changers | 310 | 310 |
| Cohabiting - Black | -0.001 | -0.004 |
|  | [0.005] | [0.007] |
| N - mother-year | 2,595 | 2,624 |
| N - changers | 766 | 765 |
| Cohabiting - Hispanic | -0.013** | 0.006 |
|  | [0.006] | [0.008] |
| N - mother-year | 1,865 | 1,922 |
| N - changers | 587 | 587 |
| Single - White | -0.009 | -0.000 |
|  | [0.012] | [0.014] |
| N - mother-year | 668 | 655 |
| N - changers | 195 | 195 |
| Single - Black | -0.003 | -0.021*** |
|  | [0.003] | [0.006] |
| N - mother-year | 4,073 | 3,990 |
| N - changers | 1,198 | 1,192 |
| Single - Hispanic | -0.009 | -0.002 |
|  | [0.007] | [0.009] |
| N - mother-year | 1,273 | 1,281 |
| N - changers | 403 | 400 |

Note: i) The sample is pooled and includes all mothers in waves 2-5 who report information on assets; ii) Models control for wave fixed-effects and mother specific fixed-effects (see equation 2); iv) Standard errors are shown in brackets.

*** p<0.01, ** p<0.05, * p<0.1.
